# Gastroprotective Effects of S-Adenosyl-L-Methionine Through Its Antioxidant and Anti-inflammatory Properties on Dextran Sulfate Sodium-Induced Chronic Colitis in Swiss Albino Mice

**DOI:** 10.7759/cureus.86175

**Published:** 2025-06-16

**Authors:** Sanket Gaikwad, Sharmila Jalgaonkar, Raakhi Tripathi

**Affiliations:** 1 Department of Pharmacology, All India Institute of Medical Sciences (AIIMS), Nagpur, IND; 2 Department of Pharmacology and Therapeutics, Seth Gordhandas Sunderdas (GS) Medical College and King Edward Memorial (KEM) Hospital, Mumbai, IND

**Keywords:** colon weight-to-length ratio, disease activity index, glutathione (gsh), inflammatory bowel disease (ibd), tnf-α

## Abstract

Introduction: Ulcerative colitis (UC) is the most common form of inflammatory bowel disease worldwide. It is a chronic, debilitating condition that affects an individual throughout life. Patients of UC tend to have a reduced quality of life from continuous disease activity and significant complications, with a risk of developing colon cancer later in life. The current therapy does not aim to cure the disease but focuses on suppressing symptoms and improving quality of life. Despite great advances in the modern management of UC with the introduction of new effective drugs, adoption of stricter endpoints, and use of better treatment strategies, there remain many unmet needs. S-Adenosyl-L-methionine (SAMe) is available as a dietary supplement that exerts anti-inflammatory and antioxidant effects. It is also approved for add-on use in depression, osteoarthritis, and liver diseases. The present study examined the gastroprotective effects of SAMe in an animal model that simulates UC.

Materials and methods: The aim of this study was to investigate the protective effects of SAMe in an animal model of chronic colitis. After Institutional Animal Ethics Committee (IAEC) approval (IAEC/03/2022), 32 Swiss albino mice were divided into four groups with eight animals per group as the normal control (NC), disease control (DC), positive control (SLZ), and test group (SAMe). All study groups except the NC group (given normal saline) were administered 2% dextran sulfate sodium (DSS) in drinking water for seven days and only drinking water for the next seven days. This cycle of 14 days was repeated three times to induce chronic colitis. The positive control group was given sulfasalazine 100 mg/kg/day, and the test group was given SAMe 200 mg/kg/day from day 1 to day 42. The Disease Activity Index (DAI) was assessed every seven days. On day 42, all animals were sacrificed, and the colon was isolated to assess the weight-by-length ratio, macroscopic grading, histopathological scoring, and biomarkers: TNF-α and glutathione (GSH). The colon weight-by-length ratio was assessed and analyzed using one-way ANOVA. DAI, colitis macroscopy, and histopathology score were assessed and analyzed using the Kruskal-Wallis test. A value of p < 0.05 was considered to be statistically significant.

Results: It was observed that SAMe significantly reduced the colon weight-by-length ratio, colitis macroscopy grading, DAI, histopathology score, and TNF-α level as compared to the DC group, which indicated its anti-inflammatory effect. SAMe also significantly increased the GSH level as compared to the DC group, which indicated its antioxidant effect. Also, it was observed that the effects of SAMe were comparable to the positive control sulfasalazine.

Conclusion: According to our study's findings, SAMe exerts a gastroprotective effect comparable to that of sulfasalazine, as evidenced by the reduction in DAI, colon weight-by-length ratio, macroscopy grading, histopathology score, and TNF-α level. The increase in the GSH levels by SAMe signifies a decrease in oxidative stress, which may be responsible for the gastroprotective effect.

## Introduction

Inflammatory bowel disease (IBD) is a collective term for a range of clinical phenotypes caused by chronic, idiopathic, and remitting inflammation of the gastrointestinal tract. Crohn’s disease (CD) and ulcerative colitis (UC) are the two most common forms of IBD [1]. IBD is a lifelong disease with no cure [2]. Symptoms include abdominal pain, chronic and relapsing episodes of diarrhea, nausea, vomiting, weight loss, anorexia, and fatigue, significantly impacting health and quality of life [3].

UC is more prevalent than CD in India, with over 1.1 million people affected, marking the second-highest disease burden worldwide after the United States [4]. A male preponderance is also noted for UC (the ratio of male:female was 1.5:1) [5].

Although the exact etiology of UC remains unclear, substantial evidence suggests that an inappropriate immune response to environmental factors such as drugs, toxins, infections, or intestinal microbes in a genetically susceptible host contributes to disease pathogenesis. It is diagnosed clinically, supported by endoscopic findings, histopathology, and exclusion of infectious causes [6].

The primary goals of UC treatment are to induce and maintain remission, prevent complications such as colorectal cancer, and improve quality of life [7]. First-line treatment involves sulfasalazine (SLZ) and 5-aminosalicylates (5-ASA), with remission rates of ~50%. Glucocorticoids are added if remission is not achieved within two weeks, while thiopurines (azathioprine or 6-mercaptopurine) or biologics (infliximab, adalimumab) are used in steroid-refractory cases [6]. However, these therapies do not cure the disease but only suppress symptoms.

Adverse effects limit the long-term use of existing treatments. About 10% of patients on 5-ASA develop acute intolerance syndrome [8]. Corticosteroids provide rapid symptom relief but carry significant risks [9]. Primary failure of anti-TNF therapy occurs in 19%-58% of patients, and long-term remission rates remain low (~26%). Additionally, secondary loss of response to TNF inhibitors occurs in 17%-22% of patients [10]. Thiopurine’s long-term use is linked to increased risks of urinary tract and skin cancers, with a fourfold increased risk of lymphoma [11].

Given these limitations, there is a need for a treatment that induces sustained corticosteroid-free remission, provides a rapid onset of action, effectively treats moderate-to-severe disease, and reduces hospitalization and surgery rates. This led to the investigation of S-adenosyl-L-methionine (SAMe), a nutraceutical approved as an adjunct treatment for depression [12], osteoarthritis [13], and liver disease [14]. Studies indicate that SAMe levels are reduced in colitis [15]. SAMe exerts anti-inflammatory, antioxidant, and analgesic effects and influences DNA methylation [16,17].

A literature review revealed no prior studies evaluating SAMe in chronic colitis [14]. Previous research in pentylenetetrazole (PTZ)-induced kindling and Aβ-induced neuroinflammation in rats used 10-250 mg/kg SAMe, demonstrating antioxidant properties [18,19]. Based on these findings, a 200 mg/kg dose was chosen for this study as a clinically relevant dose.

Dextran sulfate sodium (DSS) is widely used to induce colitis in rodents, as it mimics UC-like pathogenesis [20]. The DSS model’s relevance in translating animal findings to human colitis makes it ideal for evaluating SAMe’s protective effects in chronic colitis. This study aims to investigate the protective effects of SAMe in DSS-induced chronic colitis in mice and to elucidate its molecular mechanisms.

## Materials and methods

After receiving Institutional Animal Ethics Committee approval (IAEC/03/2022), the study was conducted according to the Committee for the Control and Supervision of Experiments on Animals (CCSEA) guidelines.

Study drugs/chemicals

The test drug, SAMe, was procured from The Bangalore Sales Corporation. SLZ, used as the positive control, was procured from Sigma Aldrich. The inducing agent, DSS, was procured from SRL Laboratories Pvt. Ltd., India.

Experimental animals

Animals randomly bred in the Center for Animal Studies of the Institute were used. Thirty-two Swiss albino mice of either sex aged 6-8 weeks and weighing 18-22 g were used in this study. Animals had free access to filtered water and feed in the form of pellets. Regulated conditions were maintained with temperature 18°C-29°C, humidity 30%-70%, and a 12-hour light-dark cycle.

Experimental design

Animals were divided into four groups of eight mice each group as follows (Table 1).

**Table 1:** Details of the study groups, dose, duration, and route of study drugs and inducing agent DSS: dextran sulfate sodium

Group (n = 8)	Drug/vehicle (from day 1 to day 42)	Dose (oral)	Inducing agent (from day 1 to day 7, day 15 to day 21, & day 29 to day 35)
Normal control (NC)	Normal drinking water		
Disease control (DC)	Normal saline	1 mL	2% DSS
Positive control (SLZ)	Sulfasalazine	100 mg/kg	
Test drug (SAMe)	S-Adenosyl-L-methionine	200 mg/kg	

The normal control (NC) group received only normal drinking water. The disease control (DC) group received 2% DSS dissolved in drinking water from day 1 to day 7 followed by only drinking water from day 8 to day 14. This cycle of 14 days was repeated three times to induce chronic colitis [21]. The positive control (SLZ) group received 2% DSS as explained above and SLZ orally by feeding tube in the dose of 100 mg/kg daily from day 1 to day 42 [22]. The test drug (SAMe) group received 2% DSS as explained above along with SAMe 200 mg/kg orally by feeding tube daily from day 1 to day 42 [18,19].

Experimental procedure

**Figure 1:**
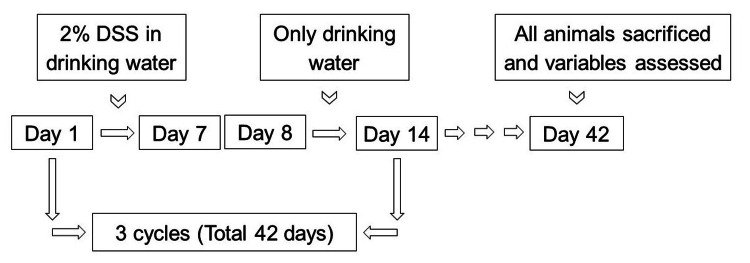
Experimental procedure showing three cycles of 2% DSS in drinking water for seven days & only drinking water for the next seven days DSS: dextran sulfate sodium

Figure 1 depicts the experimental procedure.

Preparation of 2% DSS

Two grams of DSS was measured using a digital weighing scale. It was then dissolved in 100 mL of drinking water. The solution was then poured into the sipper bottle, and the bottle was kept in a cage containing mice. The drinking water was changed daily, and a fresh 2% DSS solution was prepared each day.

The variables assessed include the Disease Activity Index (DAI) on days 7, 14, 21, 28, 35, and 42; the colon weight-by-length ratio on day 42; macroscopic grading of colitis on day 42; histopathological examination on day 42; and biomarkers (TNF-α and glutathione (GSH)) on day 42.

Measurement of DAI

The DAI for each animal was calculated on days 7, 14, 21, 28, 35, and 42 by summing up the score of three variables (weight loss, stool consistency, and blood in stool), and the mean DAI of a group for that day was determined [23].

Measurement of the colon weight-by-length ratio (mg/cm)

The isolated colon was rinsed with a sterile phosphate buffer solution (PBS) to clear the fecal matter [24]. The length and the weight of each colon were measured using a Vernier caliper and a digital weighing scale, respectively, and the colon weight-by-length ratio was calculated.

Macroscopic grading of colitis

The colons were longitudinally incised and examined for the presence of ulcers, and the severity of colitis was assessed macroscopically using a previously established scoring system [24].

Histopathological examination

The longitudinally incised colons of each mouse were individually rolled using the “Swiss roll technique” [24]. The rolled colon samples were fixed, embedded in liquid paraffin, sectioned, and stained with hematoxylin and eosin (H&E). The sections were microscopically examined for histopathologic changes using a validated scoring system as described previously [23]. The histopathology score was determined by multiplying the sum of the scores of the three histological features by the score for the percent area of involvement. Thus, the minimum and maximum scores that could be obtained were 0 and 40, respectively.

Biochemical marker estimation: TNF-α and GSH in colon tissue

The proximal part of the colon was thoroughly washed in PBS to remove excess blood. The colonic tissue was homogenized in PBS on ice. The homogenized tissues were then centrifuged at 3,000 RPM in a refrigerated centrifuge for approximately 20 minutes. The supernatant was collected in Eppendorf tubes using micropipettes and stored at -80°C for estimation of TNF-α and GSH levels by enzyme-linked immunosorbent assay (ELISA).

Statistical analysis

All the results were expressed as the mean ± standard deviation (SD). Data analysis was performed using GraphPad InStat V3.06 software (Dotmatics, Boston, MA, US). Data of parametric variables (colon weight-by-length and colonic TNF-α and GSH levels) were analyzed using one-way ANOVA followed by Dunnett’s post hoc analysis. The non-parametric variables (DAI, colitis macroscopic score, and colitis histology score) were analyzed by either the Kruskal-Wallis test followed by the post hoc Dunn test or the Mann-Whitney test. A value of p < 0.05 was considered to be statistically significant.

## Results

There was a mortality of two mice in the DC group. Hence, the results presented are with six animals for the DC group. Meanwhile, for the NC, SLZ, and SAMe groups, the results presented are for eight animals per group.

Variables assessed

Disease Activity Index

**Figure 2:**
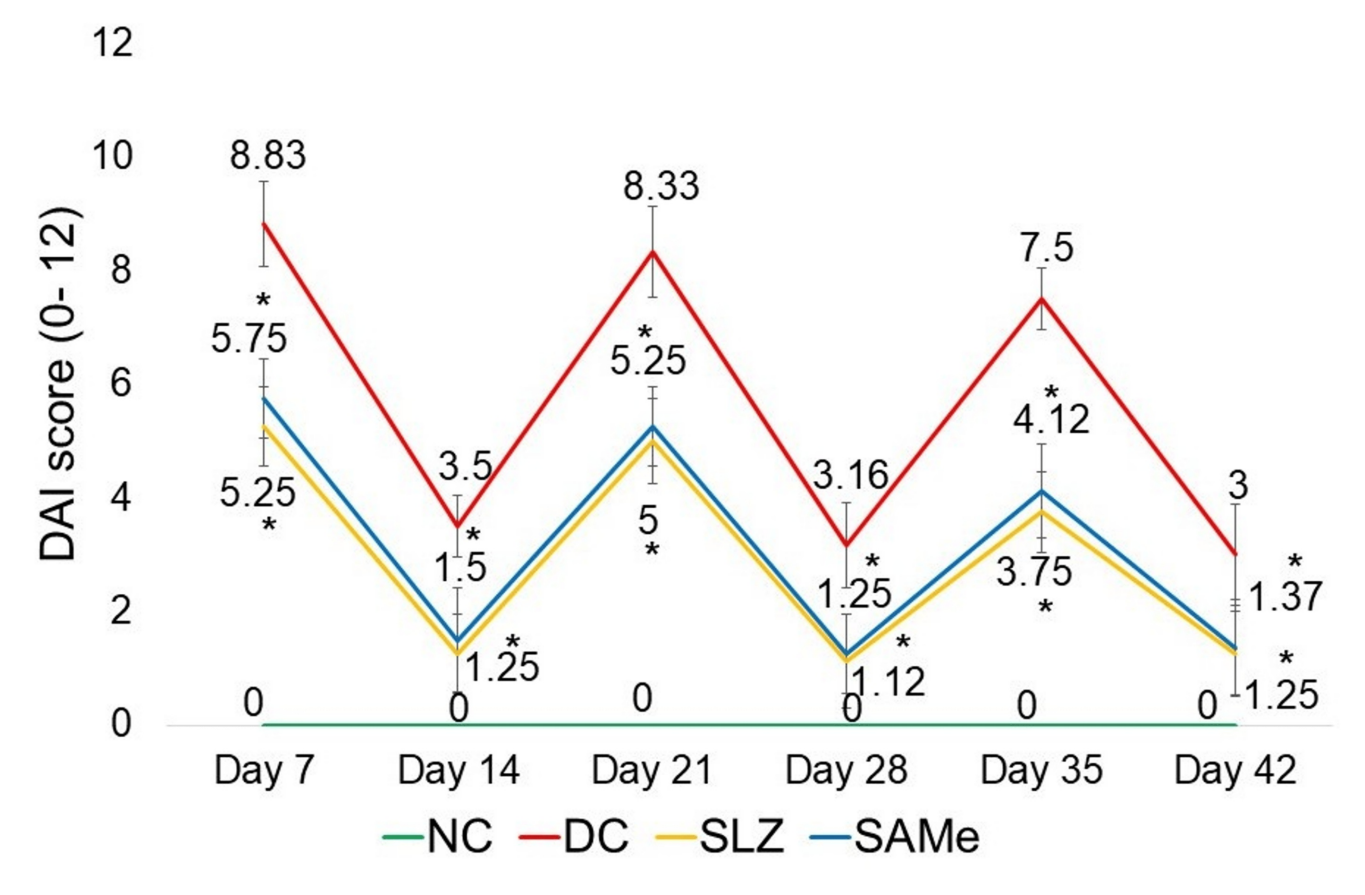
DAI in different experimental groups NC: normal control (n = 8); DC: disease control (n = 6); SLZ: positive control (sulfasalazine, n = 8); SAMe: test drug (S-adenosyl-L-methionine, n = 8); DAI: Disease Activity Index; SD: standard deviation. Mean ± SD. Kruskal-Wallis test followed by the post hoc Dunn test. *p < 0.05 vs. DC

The DAI scores shown by the SLZ group on days 7, 14, 21, 28, 35, and 42 were significantly lower (p < 0.05) than that of the DC group. Treatment with the SAMe significantly reduced (p < 0.05) the DAI as compared to the DC. The DAI score for the SAMe group was comparable to the SLZ group for all days (Figure 2).

Colon Weight-by-Length Ratio

**Figure 3:**
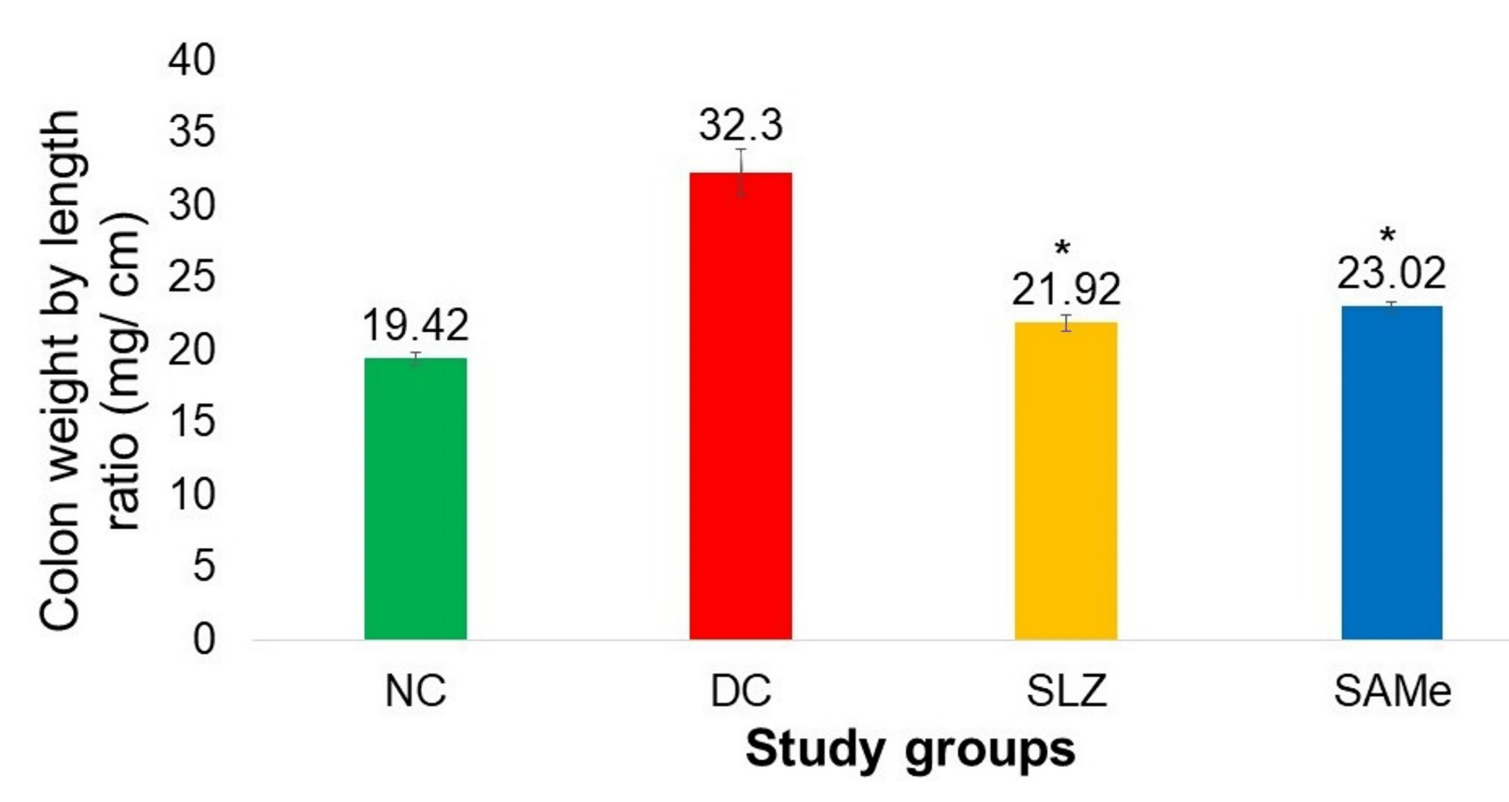
Colon weight-by-length ratio in different experimental groups NC: normal control (n = 8); DC: disease control (n = 6); SLZ: positive control (sulfasalazine, n = 8); SAMe: test drug (S-adenosyl-L-methionine, n = 8); SD: standard deviation. Mean ± SD. The ANOVA test followed by post hoc Dunnett’s test. *p < 0.05 vs. DC

There was a significantly low colon weight-by-length ratio in the SAMe group compared to the DC group (23.02 ± 0.32 vs. 32.3 ± 1.61; p < 0.05). There was no significant difference in the colon weight-by-length ratio in the SAMe group when compared to SLZ (23.02 ± 0.32 vs. 21.92 ± 0.58; p < 0.05 vs. DC group) (Figure 3).

Macroscopic Grading of Colitis

**Figure 4:**
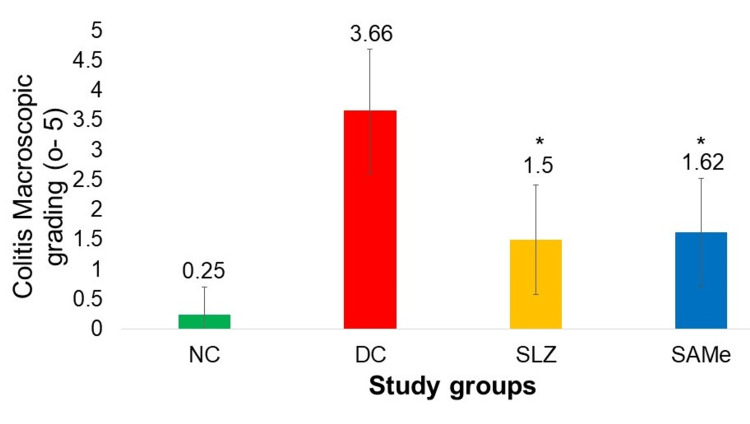
Macroscopic grades of colitis in different experimental groups NC: normal control (n = 8); DC: disease control (n = 6); SLZ: positive control (sulfasalazine, n = 8); SAMe: test drug (S-adenosyl-L-methionine, n = 8); SD: standard deviation. Mean ± SD. Kruskal-Wallis test followed by the post hoc Dunn test. *p < 0.05 vs. DC

SAMe showed a significantly low macroscopic grading score (1.62 ± 0.91 vs. 3.66 ± 1.03; p < 0.05) as compared to DC, and these scores were comparable to SLZ (1.62 ± 0.91 vs. 1.5 ± 0.92; p < 0.05 vs. DC group) (Figure 4).

Colon Histopathology Score

**Figure 5:**
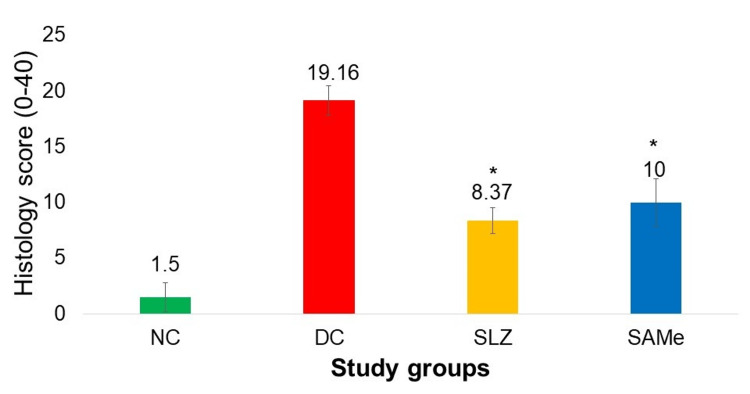
Colon histopathology score in various experimental groups NC: normal control (n = 8); DC: disease control (n = 6); SLZ: positive control (sulfasalazine, n = 8); SAMe: test drug (S-adenosyl-L-methionine, n = 8); SD: standard deviation. Mean ± SD. Kruskal-Wallis test followed by the post hoc Dunn test. *p < 0.05 vs. DC

**Figure 6:**
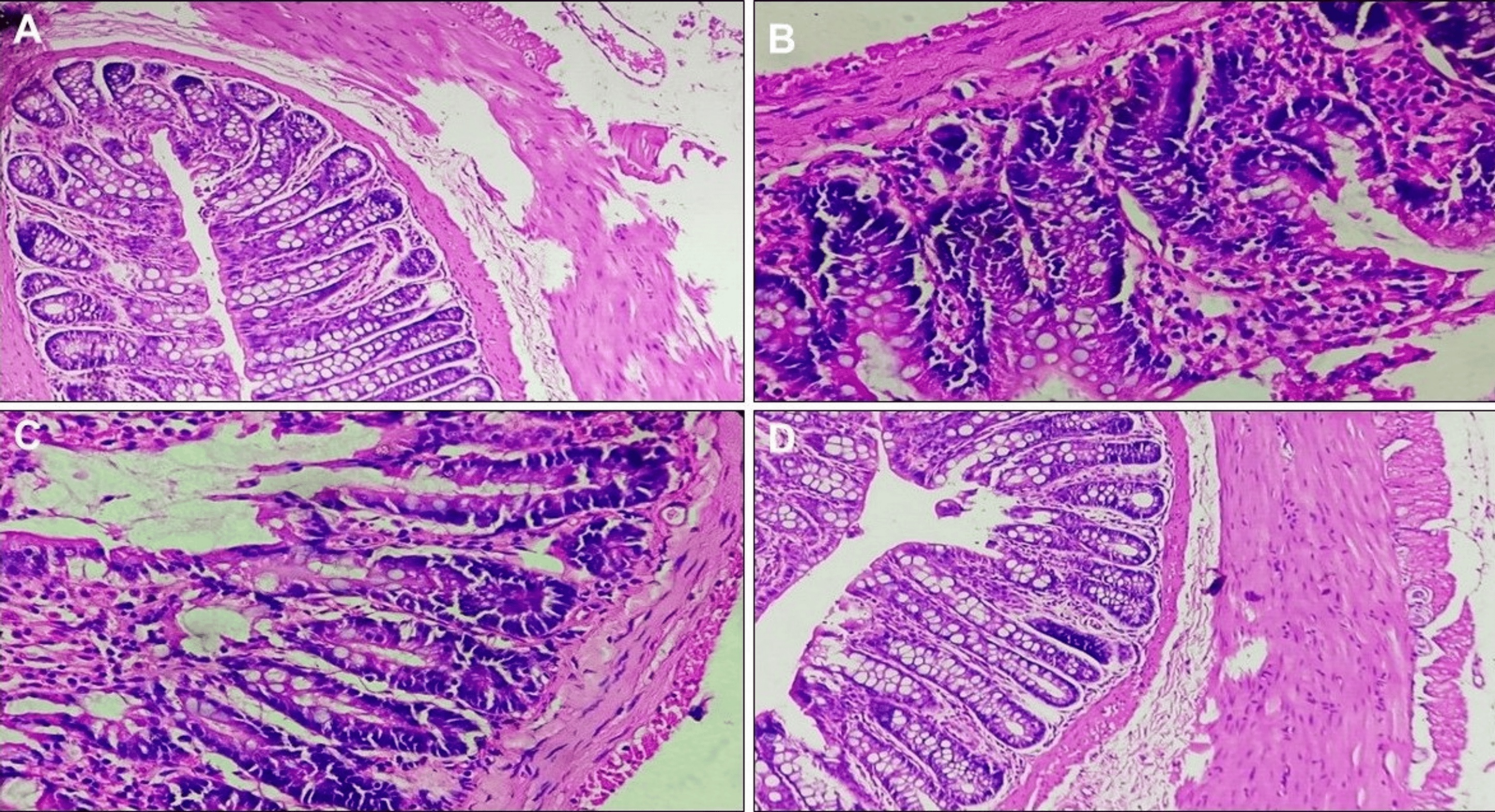
Histopathology images of the colon (stained with H&E and viewed at 400x magnification) (A) Normal colon with normal crypt architecture & intact epithelial lining; (B) diseased colon with completely damaged crypts along with epithelial erosion and dense lymphocytic infiltration; (C) positive control, sulfasalazine-treated colon showing near-normal crypts with minimal mucosal lymphocyte infiltration; (D) SAMe-treated colon showing near-normal crypt architecture with minimal mucosal lymphocyte infiltration H&E: hematoxylin and eosin

The histology scores of the group that received SAMe were found to be significantly lower as compared to the DC group (10 ± 2.13 vs. 19.16 ± 1.32; p < 0.05). These scores for SAMe were not significantly different from the group that received SLZ (10 ± 2.13 vs. 8.37 ± 1.18; p < 0.05 vs. DC group) (Figures 5, 6).

Biomarkers: Colonic TNF-α and GSH Levels

**Figure 7:**
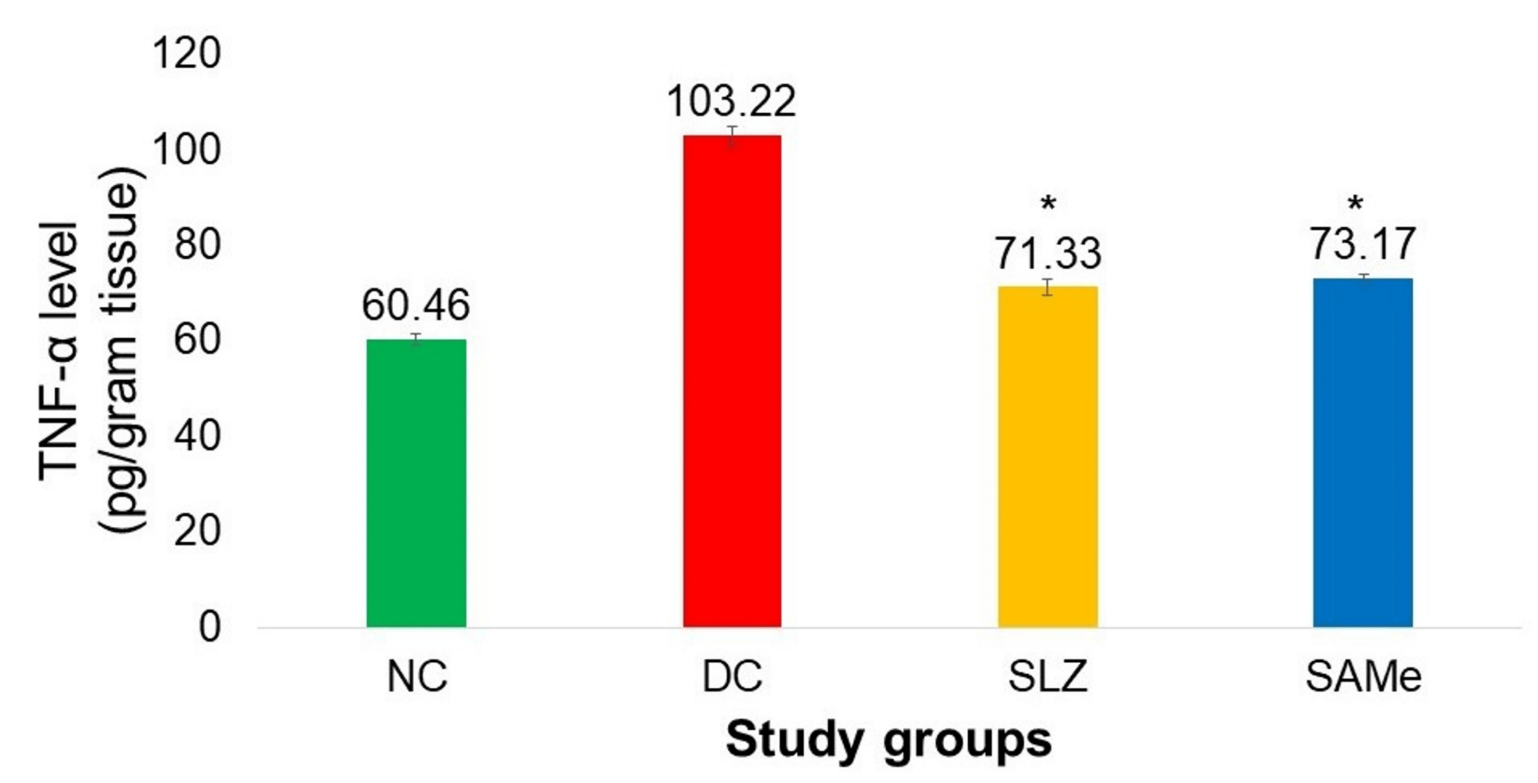
Colonic TNF-α levels (pg/g tissue) NC: normal control (n = 8); DC: disease control (n = 6); SLZ: positive control (sulfasalazine, n = 8); SAMe: test drug (S-adenosyl-L-methionine, n = 8); SD: standard deviation. Mean ± SD. ANOVA test followed by post hoc Dunnett’s test. *p < 0.05 vs. DC

**Figure 8:**
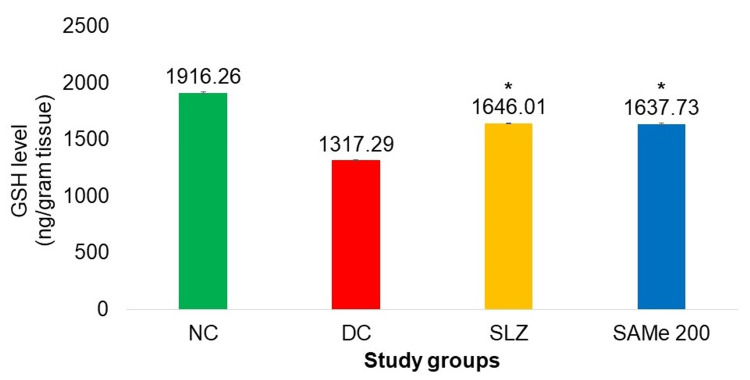
Colonic GSH level (ng/g tissue) NC: normal control (n = 8); DC: disease control (n = 6); SLZ: positive control (sulfasalazine, n = 8); SAMe: test drug (S-adenosyl-L-methionine, n = 8); SD: standard deviation; GSH: glutathione. Mean ± SD. ANOVA test followed by post hoc Dunnett’s test. *p < 0.05 vs. DC

The TNF-α level in the colon was significantly lower in the group that received SAMe as compared to the DC group (SAMe: 73.17 ± 0.79 vs. 103.22 ± 1.87; p < 0.05). The GSH level in the colon was significantly higher in SAMe compared to the DC group (1,637.730 ± 7.09 vs. 1,317.29 ± 5.43; p < 0.05). For both of these biochemical variables, improvements seen in the SAMe group were comparable to the SLZ group (Figures 7, 8).

## Discussion

This study evaluated the anti-inflammatory, antioxidant, and cytoprotective properties of SAMe in a DSS-induced chronic colitis model. The results indicate that SAMe was successful in reducing inflammation. There was a significant difference between the DAI, colon weight-by-length ratio, macroscopic colitis grading, histopathology score, and biomarkers TNF-α and GSH of the SAMe-treated group and the disease-control group.

Inflammation and oxidative damage are important pathophysiological features of UC [25]. In a study by Schmedes et al., low levels of SAMe were found in patients with severe IBD [15]. Li et al. have shown that endogenous SAMe plays an important role in intestinal homeostasis and prevents inflammation [26]. This study also showed the beneficial role of SAMe in reducing colitis by decreasing inflammation; however, the study was conducted using a nine-day colitis model, and there was no comparison with the standard of care [26]. There are no studies of SAMe in chronic colitis models. Hence, SAMe was evaluated for chronic colitis.

According to various clinical guidelines, SLZ is a first-line drug for managing patients with UC [27]. SLZ has been employed as a comparator in numerous pre-clinical studies evaluating the effects of test drugs on acute and chronic colitis in mice, all of which used the same dose of 100 mg/kg/day, the dose also used in our study [28,29].

Among the numerous animal models that have been developed, chemically induced models of IBD are most commonly used because of their simplicity, controllability, and low cost. The DSS model of colitis is more flexible than other chemically induced animal models of colitis. Researchers can produce acute, chronic, or relapsing models by changing the concentration and dosing frequency of DSS administration. Mizoguchi suggested a chronic DSS model if the drug targets are TNF-α and interferon-γ [30].

The doses and duration used for the induction of chronic colitis vary in the literature. Hoffmann et al. used 2% DSS for the first seven days, followed by 1% DSS for 10 days and 2% DSS for another 12 days to induce chronic colitis [31]. Wirtz et al. suggested the use of three cycles of 2%-3% DSS (seven days of DSS induction, 14 days of water) to induce chronic colitis [32]. Kwon et al. induced chronic colitis using three cycles of 2% DSS for seven days and only drinking water for the next seven days [21]. It was found in the literature that the most commonly used concentration of DSS was 2% to induce chronic colitis. Therefore, we decided to induce chronic colitis with three cycles of 2% DSS for seven days and only drinking water for the next seven days, as used by Kwon et al. Swiss Albino mice were a choice of species as the human gut microbiomes are functionally similar to theirs [33].

As seen from the results, there was a significant increase in the DAI scores of the DC group seen on days corresponding to the DSS cycle (day 7: 7.75 ± 0.70, day 21: 5.87 ± 0.83, and day 35: 5.25 ± 0.70). DAI on days in between, i.e., days 14 (2 ± 0.75), 28 (3.25 ± 0.70), and 42 (2.37 ± 0.91), remained lower during drinking water cycle days. The observed pattern of alternating exacerbation and remission, as seen in our study, is consistent with the episodic nature of chronic colitis, demonstrating the ongoing inflammation and cyclical symptom severity typical of UC. These findings are similar to the ones seen in other studies that have used the DSS-induced experimental model of chronic colitis [21,31,32,34].

The group that received SAMe showed significant improvement in all variables compared to DC, i.e., DAI from day 7 to day 42, colon weight-by-length ratio, colon macroscopic grading, and histopathological grading. There was also improvement in biomarkers compared to DC, with the reduction in TNF-α (73.17 ± 0.79 vs. 103.22 ± 1.87) and increased GSH (1,637.730 ± 7.09 vs. 1,317.29 ± 5.43) levels.

DAI in SAMe-treated mice was significantly lower than that in the DC group (day 7: 5.75 ± 0.7 vs. 8.83 ± 0.75, day 21: 5.25 ± 0.27 vs. 8.33 ± 0.81, and day 35: 4.12 ± 0.83 vs. 7.5 ± 0.54). The shortening of the colon length and an increase in colon weight are indicators of edema and inflammation in the colon. Reduction of the colon weight-by-length ratio by SAMe indicated that it prevented colon shortening and improved colon weight, thus decreasing the severity of DSS-induced colitis compared to DC (23.02 ± 0.32 vs. 32.3 ± 1.61).

DSS induces intestinal inflammation and is associated with disrupting the intestinal epithelial monolayer lining. The macroscopic and histological features in DC mouse colons in our study are the same as those reported by Xu et al. [35]. SAMe showed improvement in macroscopic (1.62 ± 0.91 vs. 3.66 ± 1.03) and histological (10 ± 2.13 vs. 19.16 ± 1.32) gradings compared to DC results similar to Li et al. [26]. In this study, SAMe group addition led to less crypt damage, a slight decrease of ulceration, and a subtle reduction of inflammatory infiltration compared to vehicle.

Apart from colon length and histopathology variables used in Li et al.'s study [26], additional important variables used in our study were colon tissue TNF-α and GSH levels. When colonic levels of TNF-α were estimated, it was observed that SAMe lowered the TNF-α levels significantly as compared to DC. The reduction in TNF-α caused by SAMe was comparable to SLZ. Oz et al. [25] assessed the serum level of TNF-α and found that SAMe significantly reduced the serum TNF-α in DSS-treated mice compared to the DC. However, they did not compare the effects of SAMe with SLZ.

Oxidative stress is a key factor in the pathogenesis of intestinal damage in UC. It has been suggested that the low concentrations of endogenous antioxidants, such as GSH peroxidase (GSH-Px) and superoxide dismutases (SODs), are linked to the intestinal damage observed in IBD [36]. SAMe has proven antioxidant activity as it is the precursor of GSH, a major cellular antioxidant [37]. When colonic levels of GSH were estimated, it was observed that GSH levels were significantly increased in the group that received SAMe as compared to DC. The increase in the GSH level caused by SAMe was comparable to SLZ.

In a study by Oz et al. [25], body weight and colon length were significantly reduced in the treated group. Serum TNF-α was significantly lower in the treated group. However, there was no significant difference in colonic GSH of DSS-treated mice and DSS + SAMe-treated mice, while the blood level of GSH was decreased in DSS-treated but was restored to normal levels in SAMe-treated mice. The beneficial effect of SAMe on TNF-α is due to the downregulation of genes of this proinflammatory biomarker. A similar effect on proinflammatory cytokine genes was shown by Li et al. [38], who evaluated the effect of SAMe in DSS-induced colon cancer.

Our study's results align with those drawn from the study by Li et al. [26]. After SAMe treatment in DSS-induced colitis, this study showed increased colon length, reduced body weight ratio, less crypt damage, and a slight decrease of ulceration with a reduction of inflammatory infiltrate, as shown from histopathology.

The present study is the first study to examine the effects of SAMe in an experimental model of chronic colitis. One of the strengths of this study was the assessment of the symptomatic score of DAI along with the colon-based variables, as well as the exploration of the mechanism of action of SAMe through biomarker assessment. Thus, our study, though preliminary, involved multiple variables for evaluation, both subjective and objective, to get a comprehensive picture. Conducting clinical trials concurrently with animal studies to validate our findings in UC patients would be interesting.

## Conclusions

SAMe showed a gastroprotective effect and was comparable to the standard dose of SLZ in DSS-induced chronic colitis. SAMe in DSS-induced chronic colitis resulted in a decrease in the colon weight-by-length ratio, DAI, macroscopic grading, and histopathology score. It reduced the TNF-α level and increased the GSH level in colonic tissue. The study results concluded that SAMe reduces colonic damage in DSS-induced chronic colitis due to its ability to reduce proinflammatory cytokines and increase protective antioxidants. This indicates that SAMe may have the potential to be used as a gastroprotective agent in patients with colitis.

Further animal research should be performed with other IBD biomarkers such as calprotectin, lactoferrin, CRP, and myeloperoxidase, which are clinically used biomarkers. SAMe has a DNA methylation property and is linked to the epigenetic modulation of specific genes involved in inflammation and oxidative stress, which play essential roles in the pathogenesis of UC. Therefore, a specific biomarker, 5-methylcytosine, can be used as one of the biomarkers in further animal and clinical research of SAMe. A proof-of-concept clinical trial of SAMe as an add-on drug can be performed on a small number of patients, as this drug is already marketed as a nutraceutical and has fewer side effects.
